# The impact of deep-tier burrow systems in sediment mixing and ecosystem engineering in early Cambrian carbonate settings

**DOI:** 10.1038/srep45773

**Published:** 2017-04-04

**Authors:** Li-Jun Zhang, Yong-An Qi, Luis A. Buatois, M. Gabriela Mángano, Yao Meng, Da Li

**Affiliations:** 1 Institute of Resources and Environment, Key Laboratory of Biogenic Traces & Sedimentary Minerals of Henan Province, Collaborative Innovation Center of Coalbed Methane and Shale Gas for Central Plains Economic Region, Henan Polytechnic University, Jiaozuo 454003, P.R. China; 2 Department of Geological Sciences, University of Saskatchewan, 114 Science Place, Saskatoon, Saskatchewan S7N 5E2, Canada

## Abstract

Bioturbation plays a substantial role in sediment oxygen concentration, chemical cycling, regeneration of nutrients, microbial activity, and the rate of organic matter decomposition in modern oceans. In addition, bioturbators are ecosystem engineers which promote the presence of some organisms, while precluding others. However, the impact of bioturbation in deep time remains controversial and limited sediment mixing has been indicated for early Paleozoic seas. Our understanding of the actual impact of bioturbation early in the Phanerozoic has been hampered by the lack of detailed analysis of the functional significance of specific burrow architectures. Integration of ichnologic and sedimentologic evidence from North China shows that deep-tier *Thalassinoides* mazes occur in lower Cambrian nearshore carbonate sediments, leading to intense disruption of the primary fabric. Comparison with modern studies suggest that some of the effects of this style of Cambrian bioturbation may have included promotion of nitrogen and ammonium fluxes across the sediment-water interface, average deepening of the redox discontinuity surface, expansion of aerobic bacteria, and increase in the rate of organic matter decomposition and the regeneration of nutrients. Our study suggests that early Cambrian sediment mixing in carbonate settings may have been more significant than assumed in previous models.

Bioturbation, involving both particle and solute transport within burrows, into the surrounding sediment and across the sediment-water interface, is one of the most important factors in affecting oxygen concentration in the sediment, chemical cycling, regeneration of nutrients, microbial activity and the rate of organic matter decomposition in modern oceans^1^,^2^,^3^. In addition, burrowing animals represent ecosystem engineers that impact on community structure by either having negative (e.g., influence of infaunal deposit feeders on sessile epifaunal and infaunal suspension feeders) or positive (e.g., influence of infaunal deposit feeders on meiofauna and microbes) effects on other organisms^4^,^5^. There is considerable debate regarding the timing of infaunalization and the role that bioturbation may have played in nutrient cycling and sediment mixing during the Cambrian Explosion. Unfortunately, precise evaluation of the actual impact of bioturbation early in the Phanerozoic has been hampered by the lack of detailed analysis of the functional significance of specific burrow architectures preserved in the fossil record. Whereas most research have focused on measuring degree of bioturbation and estimating maximum burrowing depths, the actual role of specific type of burrows in sediment mixing and impact on other organisms has received much less attention. As a result, no attempts have been made in order to establish a link between the functional significance of discrete burrow architectures and their potential impact on sediment properties and the accompanying benthic faunas.

Integration of ichnologic and sedimentologic evidence from North China shows that intense bioturbation, mainly revealed by the ichnogenus *Thalassinoides*, took place in nearshore carbonate sediments during the early Cambrian, providing evidence of significant infaunalization and sediment mixing. Comparison of these burrow architectures with modern counterparts allows evaluating the role of these deep-tier bioturbators in sediment mixing, geochemical recycling and ecosystem engineering. The aims of this paper are to document the occurrence of pervasive deep-tier *Thalassinoides* bioturbation in shallow-marine carbonates of the lower Cambrian Zhushadong Formation of North China, and discuss how this style of burrowing may have affected shallow-water carbonate ecosystems and associated sediments.

## Geologic setting

The Zhushadong Formation crops out in southwestern Songshan Mountain, western Henan Province (Fig. S1), belonging to the Southern subprovince^6^,^7^. The three studied sections are quite continuous and located southwest of Zhengzhou (Figs S1–S2). Two well-preserved trilobite faunas assigned to the *Palaeolemis* Zone and *Redlichnia chinensis* Zone indicate a late early Cambrian age (Cambrian Age 4)^8^,^9^.

**Figure 1:**
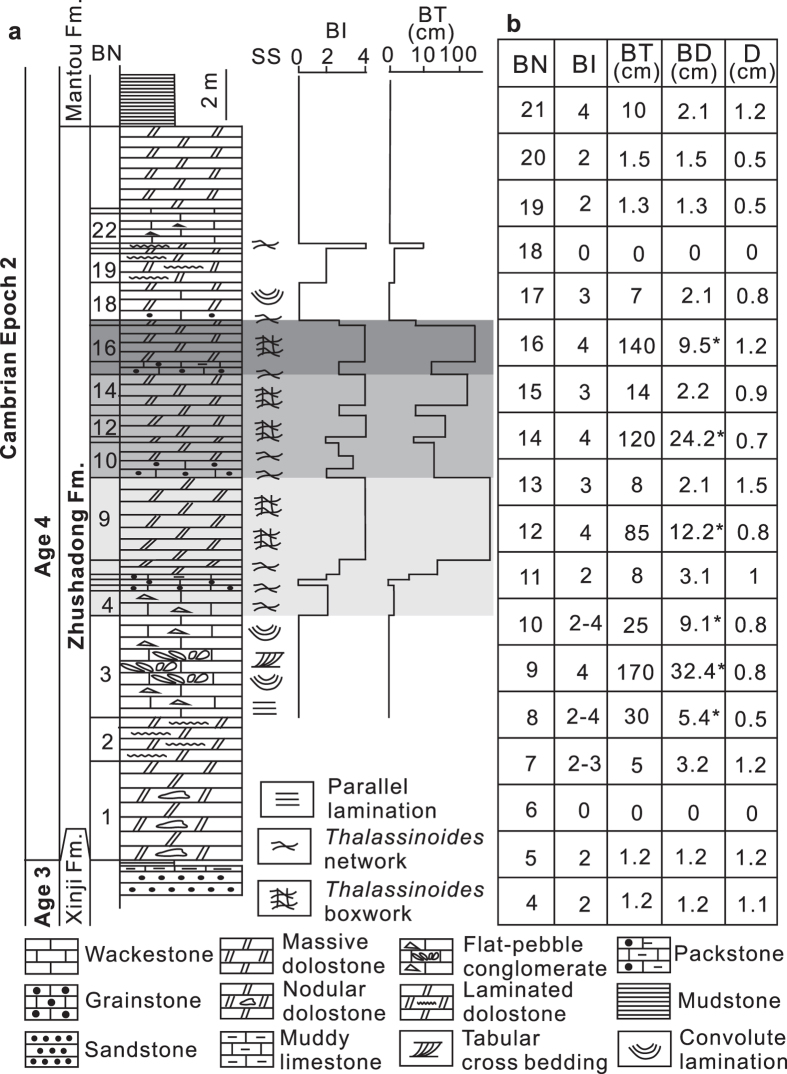
Stratigraphic column and ichnofabric data from the lower Cambrian Zhushadong Formation, North China. (**a**) Generalized stratigraphic column of the lower Cambrian Zhushadong Formation showing bioturbation index and thickness of bioturbated interval in the Guankou section from the southwestern Songshan Mountain, North China. (**b**) Table showing bioturbation index, burrow depth, thickness of bioturbated interval and diameter of *Thalassinoides* in the Zhushadong Formation, Guankou section. Abbreviations: BN = Bed number, SS = sedimentary structures, BI = bioturbation index, BT = thickness of bioturbated interval, BD = burrow depth, D = diameter of *Thalassinoides*.

The Zhushadong Formation is a 24–54-m-thick succession of dark-grey and/or light-grey, medium-thick bedded wackestone and dolostone, interbedded with packstone, grainstone and flat pebble conglomerate (Fig. 1). Stromatolites, parallel and convolute lamination are common (Figs S2–S3). Hummocky cross-stratification is present locally. The occurrence of tabular and trough cross bedding, convolute lamination and gypsum breccia^10^ suggests 2D and 3D dune migration in a restricted shallow-marine environment periodically exposed subaerially in a seasonally dry, probably subarid, climate. The influence of storm activity is suggested by the flat pebble conglomerates and the hummocky cross-stratification, whereas the soft-sediment deformation has been attributed to earthquakes^11^,^12^.

## Occurrence of *Thalassinoides* ichnofabric

**Figure 2:**
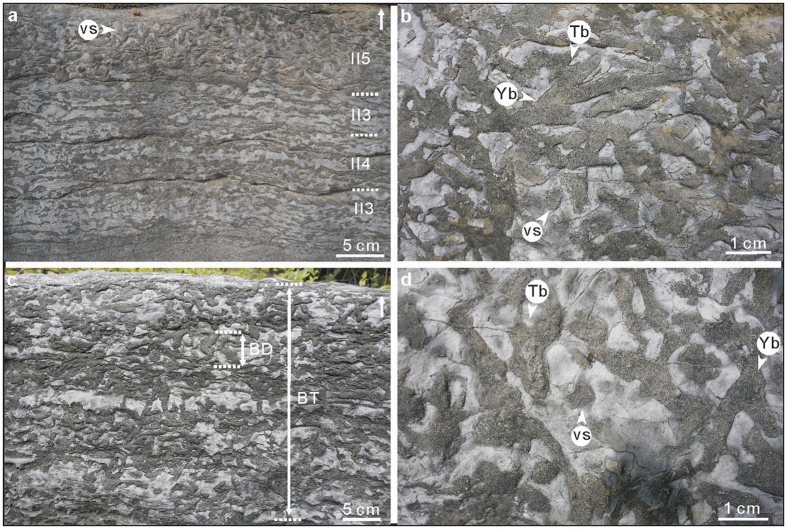
*Thalassinoides* in the restricted shallow-marine deposits of the Zhushadong Formation in the Guankou section. (**a**) General view of beds showing biooturbation indexes ranging from 2 to 4 from bottom to upper in cross-section view (Bed 8). (**b**) *Thalassinoides* network on bedding surface (Bed 9), showing three-dimensional morphology and extensive overlapping. (**c**) Strong bioturbation (BI 4) in cross-section view (Bed 9). (**d**) *Thalassinoides* network as seen on bedding surface (Bed 10), showing horizontal branches. Abbreviations: vs = vertical shaft, Tb = T-branched, Yb = Y-branched, BI = bioturbation index, BT = thickness of bioturbated interval, BD = burrow depth, *maximum burrow depth during multi-layer colonization.

**Figure 3:**
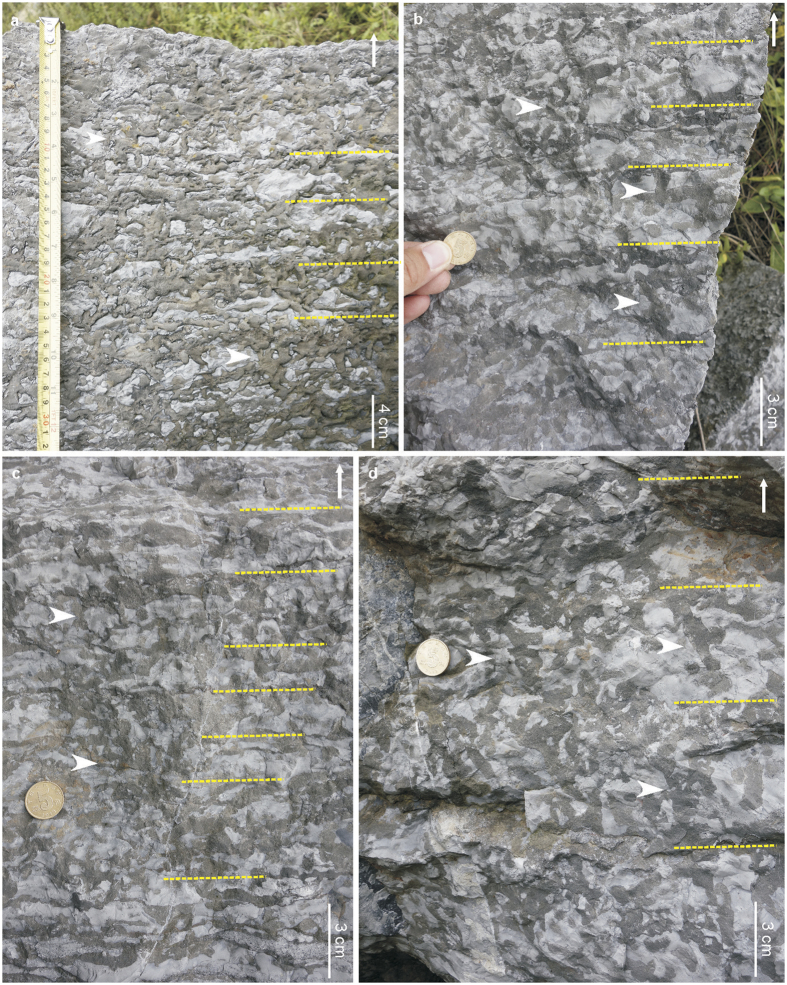
Occurrences of *Thalassinoides* representing multi-layer colonizers from the restricted platform deposits of the Cambrian Epoch 2 Zhushadong Formation in the Guankou section. (**a**) Examples from the lower part of the Zhushadong Formation (Bed 9). (**b**,**c**) Middle part of Zhushadong Formation (Bed 12). (**d**) Upper part of the Zhushadong Formation (Bed 16). *Thalassinoides* levels indicated by yellow dashed line; shafts connecting multiple layers indicated by white arrows.

Well-preserved *Thalassinoides* systems occur in 0.2–0.3 m thick dolostone beds, locally forming amalgamated units up to 3.3 m thick, from the lower to middle part of the Zhushadong Formation (Fig. 1), forming both boxworks and networks (i.e. mazes)^13^. Specimens consist of bedding-parallel polygonal networks of 0.4–1.5 cm wide, smooth, unlined (i.e. lacking wall reinforcements), mainly horizontally branching burrows (Fig. 2a–d and Table S1). Locally, short, vertical shafts are seen on bedding surfaces (Fig. 2b). In the more intensely bioturbated beds, bedding-parallel networks are linked by vertical shafts, forming a series of swollen T-junctions, delineating three-dimensional boxworks (Figs 2a and 3 and S4). Most burrows form both Y- and T-junctions but contain no swellings at junctions or elsewhere on the bedding surface. The presence of shafts distinguished the Zhushadong specimens from *Thalassinoides horizontalis* from the late Cambrian-Early Ordovician of Colorado (Fig. S4)^14^. The non-bioturbated matrix exhibits a light grey color compared to the dark grey mottled burrows. Burrow fill consists of dolomite crystals, whereas the matrix is made of micrite calcite (Fig. S5)^15^. Dolomitization linked to burrowing activity has been documented extensively in *Thalassinoides*-like burrows from the lower Paleozoic^16^,^17^.

To evaluate the degree of infaunalization in the studied Cambrian strata, we have framed our observations within the ichnofabric approach^18^,^19^. An ichnofabric refers to any aspect of the texture and internal structure of a substrate resulting from bioturbation and bioerosion at any scale^18^. To analyze the impact of this specific burrowing style on sediment mixing and on other organisms, we have measured burrow densities and established comparisons with similar burrow systems in the modern.

A total of 34 m of continuous ichnofabric data were collected from the Guankou section (Figs 1 and S2 and Table S1), 53 m in the Lushan section (Fig. S2 and Table S2), and 28 m in the Mianchi section (Figs S2 and Table S3). Most of the lower part of the Zhushadong Formation is essentially unburrowed and preserves a pristine primary fabric, characterized by algal and parallel lamination and planar cross bedding. However, intense bioturbation occurs near the top of the lower part, and in the middle and upper parts (Figs 1 and S2). Maximum penetration of individual burrow shafts is 3.1 cm, but bioturbated zones are amalgamated forming up to 170 cm thick bioturbated intervals (Fig. 1). Also, some of the shafts connect burrow systems formed at different depths, indicating that these are multi-layer colonizers^20^ able to penetrate more than one event bed. In these cases, maximum burrow depth is 32.4 cm. Beds were most likely deposited by discrete storm events. *Thalassinoides* records post-storm colonization and intense bioturbation has locally obliterated bedding boundaries, resulting in the amalgamation of bioturbated zones.

## Discussion

Although typical of post-Paleozoic rocks^21^, *Thalassinoides* is relatively common in Paleozoic strata as well^22^. *Thalassinoides* usually is interpreted as feeding burrow (Fodinichnion), typically produced by infaunal deposit feeders, such as decapod crustaceans^23^ that develop an ‘underground mining’ strategy. Early Paleozoic occurrences of this ichnogenus predate the oldest known body fossils of burrowing decapods and, therefore, other producers have been suggested^14^,^22^,^24^. Trilobites are known to have produced a wide variety of trace fossils, but their burrows rarely, if ever, have been observed to branch in an anastomosing fashion, like *Thalassinoides*. In addition, scratch marks, which are diagnostic of trilobite-produced trace fossils, have not been recorded in any of these burrow systems, further arguing against production by trilobites. The occurrence of trilobite body fossils within *Thalassinoides* has been indicated as evidence of “tunneling” behavior by these organisms^24^, but exoskeletons (exuvia or dead bodies) are commonly trapped inside burrow galleries, providing a cautionary note in establishing a genetic link between the burrow system and the preserved body fossil^25^.

Other malacostracans or even unrelated clades, such as worm-like organisms, may have been involved in the production of these structures, representing examples of behavioral convergence^14^,^22^. Within the former, phyllocarids which are known since the Cambrian and were common through the Paleozoic^26^, have been suggested as possible producers of Paleozoic *Thalassinoides*^14^,^22^. Although there is evidence that some of these crustaceans were deposit feeders which may have burrowed efficiently^26^,^27^,^28^, it is uncertain if they may have been able to produce branching burrows. Another group of shrimp-like arthropods known since the early Cambrian are waptiids^29^,^30^,^31^. Based on morphologic evidence, it has been suggested that waptiids may have been able to burrow in various type of substrate^31^, although it remains unclear if they may have produced *Thalassinoides*-like systems.

The list of Cambrian representatives of worm-like bioturbators is extensive, including priapulids^32^ and sipunculans^33^. However, these typically produced simple, unbranched burrows, rather than *Thalassinoides*-like galleries. Enteropneusts, which are known since the Cambrian^34^,^35^,^36^, are other potential candidates. Modern enteropneusts are efficient deposit-feeding bioturbators, capable of producing not only U-shaped burrows^37^,^38^,^39^, but also branching burrow systems occupying mid to deep tiers^40^. In fact, the ichnogenus *Balanoglossites* has been attributed in some cases to the work of enteropneusts^41^,^42^,^43^,^44^, resulting in structures which are remarkably similar to those referred to as *Thalassinoides* in lower Paleozoic rocks^44^. However, an epifaunal suspension-feeding mode of life has been indicated as a primitive trait^36^, therefore casting some doubts about the potential of Cambrian representatives to produce burrow systems.

Unlike the three-dimensional *Thalassinoides* (i.e. boxworks) (*circa* 1 m deep) in Upper Ordovician and younger strata^17^,^45^, *Thalassinoides* from the lower and middle Cambrian strata of the Great Basin are characterized by forming discrete small two-dimensional networks and, more rarely, three-dimensional boxworks, which produced little to negligible bioturbation^46^. The two-dimensional *Thalassinoides* continued to dominate through the Early Ordovician^14^. In fact, it is the increase in the size of *Thalassinoides* and the switch in dominance from networks to boxworks that have been deemed responsible for the increase in the extent and depth of bioturbation that took place in carbonate settings between the Middle and Late Ordovician^46^.

However, the occurrence of high-density and deep-tier *Thalassinoides* mazes penetrating several storm layers in the lower Cambrian of China suggests that these evolutionary innovations took place in proximal areas of this carbonate platform earlier than commonly assumed based on previously available information. In fact, our study suggests that deep-tier, intense bioturbation by the producers of *Thalassinoides* mazes is a consequence of the Cambrian Explosion^47^ rather than of the Great Ordovician Biodiversification Event^48^ as previously implied^46^,^47^,^48^,^49^. Invoking taphonomic or environmental reasons to explain the discrepancy between extent and depth of bioturbation between burrow systems from the Cambrian of China and occurrences elsewhere is unsupported by sedimentologic data, because a similar duration of colonization windows is expected in all these shallow-marine, storm-affected carbonate settings. In the same vein, comparable colonization windows may have resulted in broadly similar population densities of the tracemakers.

Interestingly, boxwork architectures seem to have been restricted to the proximal platform deposits represented in the Zhushadong Formation (Figs 2 and 3, S2, and S6–7), with the overlying middle to upper Cambrian Mantou and Zhangxia formations containing only shallow *Thalassinoides* networks in more open platform deposits, albeit reaching BI up to 3 (Figs S8 and S9). Therefore, information from the lower Cambrian of North China supports the model of onshore origination of evolutionary innovations and their subsequent offshore expansion^50^,^51^,^52^,^53^.

The timing of infaunalization and its consequences in terms of sediment mixing and biogeochemical cycling during the early Paleozoic is at present a matter of debate^54^,^55^,^56^,^57^,^58^. It has been recently argued that sediment mixing has been suppressed during most of the early Paleozoic being limited until the late Silurian^56^,^57^. With the exception of the classic *Skolithos* sandstone of nearshore to subtidal settings^59^,^60^,^61^, burrows tend to occupy shallow tiers in the Cambrian^54^,^56^,^57^. Because these are produced by sessile infaunal organisms (e.g., long vermiform soft-bodied animals, such as lophophorate phoronids and tantacular-crowned polychaetes^62^), their impact on sediment mixing has been typically dismissed^56^. However, other studies have indicated that the establishment of this infauna may have been instrumental in increasing the complexity of the trophic web, resulting in coupling of benthos and plankton^54^.

As with the case of *Skolithos, Thalassinoides* is produced by stationary organisms and not by sediment bulldozers. However, this type of galeries represents the activities of deep bioturbators that are able to transport sediment to the surface from below and solutes within the burrow and into the host sediment by increasing the area of sediment-water interface^1^,^2^,^3^,^63^,^64^,^65^,^66^. In modern environments, burrow systems similar to those from the Cambrian of China strongly affect nitrogen fluxes across the sediment-water interface and supply oxygen and other oxidants to microbial communities on the burrow walls^67^. This style of bioturbation induces N_2_ fixation^68^. Also, as a result of burrow construction and maintenance, ammonium is transported into the overlying water^63^. In unlined burrow systems, such as these Cambrian *Thalassinoides*, pump takes place both into and out of the sediment, further underscoring the effects of biogenic advection of porewater^69^. In addition, high densities of burrows, such as those documented here, impact on organic carbon fluxes and increase dissolved inorganic nitrogen fluxes with respect to unburrowed sediments^67^. Software ImageJ analysis used to calculate spacing among Cambrian burrows (Fig. S10 and Table S4) indicates that these are commonly within 5 mm of each other, allowing their oxygenated zone to meet even in the case of low-porosity, fine-grained sediment^70^. Experimental studies have shown that, even in the absence of significant sediment movement, the effect of burrowing is not restricted to the sediment adjacent to the bioturbator, extending instead laterally to a distance equivalent to several times the length of the producer^70^. The fact that the studied Cambrian deposits display relatively high porosities promotes even further solute transport into the surrounding sediment. In addition to solute transport, this style of bioturbation results in significant particle transport^71^.

This style of burrowing may have also played a role in promoting the presence of some organisms, while excluding others. By increasing porosity and penetrability into the substrate, bioturbation may have triggered burrowing activities by other organisms^5^. In fact, *Planolites*, a simple trace fossil attributed to deposit feeders, commonly co-occur in the muddy layers intercalated with the *Thalassinoides*-bearing beds (Fig. S7b). Also, bioturbation may have been conducive to the establishment of microbes, meiofauna and tiny animals within the burrows^5^,^71^,^72^. There is increased evidence that tiny animals^73^,^74^ and meiofauna^75^,^76^,^77^ have been important components of Cambrian marine ecosystems. On the contrary, sediment remobilization by these deposit feeders may have been detrimental for colonization of sessile suspension feeders, a phenomenon referred to as trophic group amensalism^4^,^78^. In addition, sediment turnover may have negatively impacted on larval recruitment and epifaunal grazers by diminishing microbial films on the sediment surface^5^. Neither suspension-feeder burrows, nor grazing trails have been recorded in these Cambrian carbonates.

To summarize, these bioturbators not only have significantly modified the primary sedimentary fabric, but also may have efficiently contributed to nutrient recycling, acting as ecosystem engineers as well. Some of the effects of bioturbation may have included promotion of nitrogen and ammonium fluxes across the sediment-water interface, average deepening of the redox discontinuity surface, expansion of aerobic bacteria, and increase in the rate of organic matter decomposition and the regeneration of nutrients. Our analysis suggests that sediment mixing in early Cambrian proximal shallow-marine carbonate settings may have been more intense than previously assumed. This is consistent with the results of recent global studies based on comprehensive ichnologic compilations^54^ and geochemical analysis^55^ through this critical interval of the history of the biosphere.

## Methods

Bed-by-bed sedimentologic logging was performed in three sections, Guankou, Lushan, and Mianchi. Facies analysis follows the standard practice of describing lithology, sedimentary structures, bed geometry, bed contacts and fossil content, followed by interpretation in terms of depositional processes and sedimentary environments. Ichnologic analysis involves trace-fossil sampling, study of density, abundance and distribution of biogenic structures; measurement of degree of bioturbation; and relationships among trace fossils, physical sedimentary structures, and bedding types in each sedimentary layer. Detailed maps and photographic panels of the ichnofossil-bearing strata were prepared. Software ImageJ analysis was used to calculate spacing among burrows. Thin sections were produced in order to analyze burrow fills and walls.

To assess degree of bioturbation, bioturbation index (BI)^19^ was used. This index measures the extent to which the primary sedimentary fabric is still visible. BI = 0 represents no bioturbation (0%), and a pristinely preserved primary fabric. BI = 1 (1–4%) characterized sparse bioturbation with few discrete biogenic structures locally overprinting the well-preserved sedimentary fabric. BI = 2 (5–30%) is typified by low bioturbation in sediment with well-preserved sedimentary structures. BI = 3 (31–60%) refers to sediment with discrete trace fossils, moderate bioturbation and still distinguishable bedding contacts. BI = 4 (61–90%) is characteristic of intense bioturbation, high trace-fossil density, common overlap trace fossils, and primary sedimentary structures mostly erased. BI = 5 (91–99%) represents sediment having intense bioturbation and a completely disturbed bedding. BI = 6 (100%) comprises completely bioturbated and reworked sediment, revealed by repeated overprinting of trace fossils. Bioturbation index was assessed for each bed.

Tier classification is based on current schemes^54^. The shallow tier is represented by structures emplaced in the upper 6 cm of the substrate, the mid tier comprises those produced within 6–12 cm of the substrate, the deep tier consists of those formed below 12–100 cm, and the ultra-deep tier is represented by those traces emplaced below 100 cm. The 6-cm boundary is taken to approximately coincide with a depth above which organisms are challenged by environmental disturbance instead of by maintaining contact with the sediment-water interface; below this boundary, these difficulties are reversed in severity. Below the 12-cm boundary, stresses linked to limited food supply and oxygen content, and increased substrate compaction are the dominant limiting factors. Structures produced below 100 cm are virtually unknown prior to the Mesozoic Marine Revolution^27^.

## Additional Information

**How to cite this article:** Zhang, L.-J. *et al*. The impact of deep-tier burrow systems in sediment mixing and ecosystem engineering in early Cambrian carbonate settings. *Sci. Rep.* **7**, 45773; doi: 10.1038/srep45773 (2017).

**Publisher's note:** Springer Nature remains neutral with regard to jurisdictional claims in published maps and institutional affiliations.

## Supplementary Material

Supplementary Information
