# Supplementary material for: The impact of deep-tier burrow systems in sediment mixing and ecosystem engineering in early Cambrian carbonate settings
Source: Sci Rep. 2017 Apr 4;7:45773. doi: 10.1038/srep45773 (PMC5379565; doi:10.1038/srep45773)
Supplement: Supplementary Information [file srep45773-s1.pdf]

Supplementary Materials for

The impact of deep-tier burrow systems in sediment mixing and ecosystem engineering in early Cambrian carbonate settings

Li-Jun Zhang[1], Yong-An Qi[1], Luis A. Buatois[2*], M. Gabriela Mángano[2], Yao Meng[1], & Da Li[1]

[*]To whom correspondence should be addressed. E-mail: luis.buatois@usask.ca

This PDF file includes:

Figures S1–10

Table S1–4

References

List of supplementary materials:

Figure S1. Sketch map showing location of the study area in North China.

Figure S2. Generalized stratigraphic correlation of the Cambrian Epoch 2 Zhushadong Formation, showing ichnofabric index and thickness of bioturbated interval in the Guankou, Lushan and Mianchi sections, western Henan, North China.

Figure S3. Representative sedimentary structures from the Cambrian Epoch 2 Zhushadong Formation in the Guankou section.

Figure S4. Illustration showing the basic morphology of *Thalassinoides* from the Cambrian of China and similar lower Paleozoic forms elsewhere.

Figure S5. Thin-section photographs of *Thalassinoides* in the Cambrian Epoch 2 Zhushadong Formation, Guankou section.

Figure S6. *Thalassinoides* from the restricted platform deposits of the Cambrian Epoch 2 Zhushadong Formation in the Lushan section.

Figure S7. *Thalassinoides* from the restricted platform deposits of the Cambrian Epoch 2 Zhushadong Formation in the Mianchi section.

Figure S8. Generalized stratigraphic column of the Cambrian Epoch 2–3 Zhangxia Formation, showing the bioturbation index and depth of bioturbation in the Gankou section, North China.

Figure S9. Trace fossils in the open platform deposits from the Zhangxia Formation in the Guankou section.

Figure S10. Sketch showing the steps for calculating the spacing between *Thalassinoides* based on the software ImageJ.

Table S1. Bioturbation index, thickness of bioturbated interval, burrow depth, and diameter of *Thalassinoides* in the Cambrian Epoch 2 Zhushadong Formation, Guankou section.

Table S2. Bioturbation index, thickness of bioturbated interval, burrow depth, and diameter of *Thalassinoides* in the Cambrian Epoch 2 Zhushadong Formation, Lushan section.

Table S3. Bioturbation index, thickness of bioturbated interval, burrow depth, and diameter of *Thalassinoides* in the Cambrian Epoch 2 Zhushadong Formation, Mianchi section.

Table S4. Spacing distance between *Thalassinoides* in the Cambrian Epoch 2 Zhushadong Formation, western Henan, North China.

References

Figure S1. Sketch map showing location of the study area in North China. Abbreviations: NCB = North China Block, SCB = South China Block, TB = Tarim Block, c = Guankou section (34°33′22.61″N, 113°4′52.06″E), d = Lushan section (33°48′21.47″N, 112°58′4.42″E), e = Mianchi section (34°49′36.56″N, 111°53′30.98″E). (**a**) was redrawn based on figure 1A in ref.1 and designed by Li-Jun Zhang using CorelDRAW X4 software. (b) was redrawn based on figure 2 in ref. 2 and designed by Li-Jun Zhang using CorelDRAW X4 software.

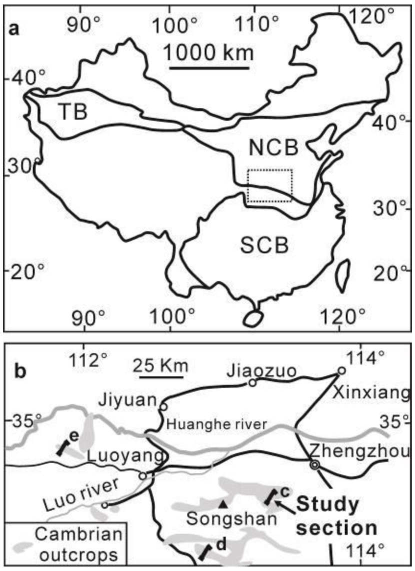

Figure S2. **Generalized stratigraphic correlation of the Cambrian Epoch 2 Zhushadong Formation, showing ichnofabric index and thickness of bioturbated interval in the Guankou, Lushan and Mianchi sections, western Henan, North China.** Abbreviations: BN = Bed number, SS = sedimentary structures, BI = bioturbation index, BT = thickness of bioturbated interval.

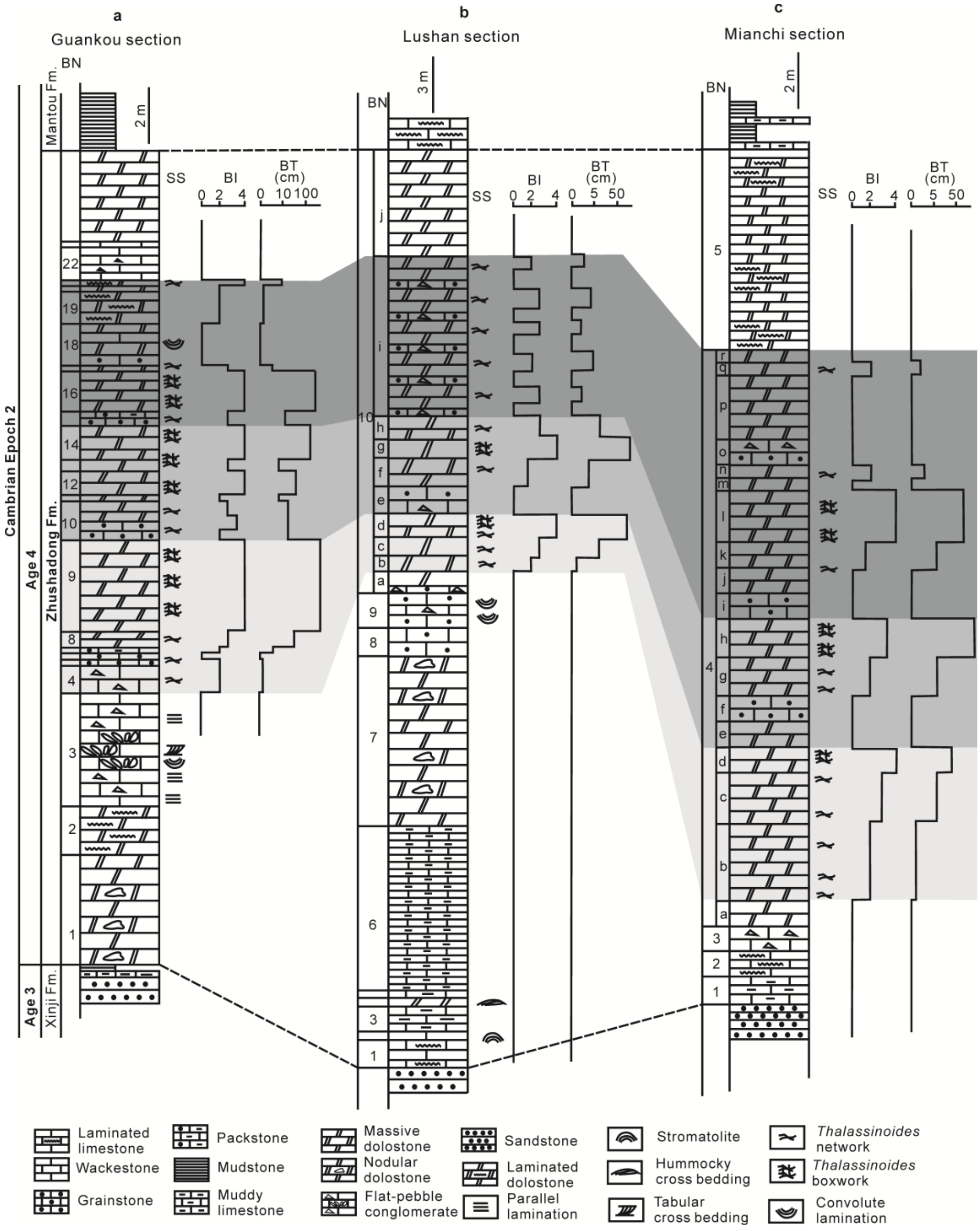

Figure S3. Representative sedimentary structures from the Cambrian Epoch 2 Zhushadong Formation in the Guankou section. (**a**) Convolute lamination in Bed 3^3. (**b**) Close-up of a. (**c**) Parallel stratification (PS) passing upwards into tabular cross bedding (CS) and trough cross stratification (TC). (**d**) Stromatolites (St). (**e**) Flat-pebble conglomerate. (**f**) Flat-pebble conglomerate and convolute lamination (CL).

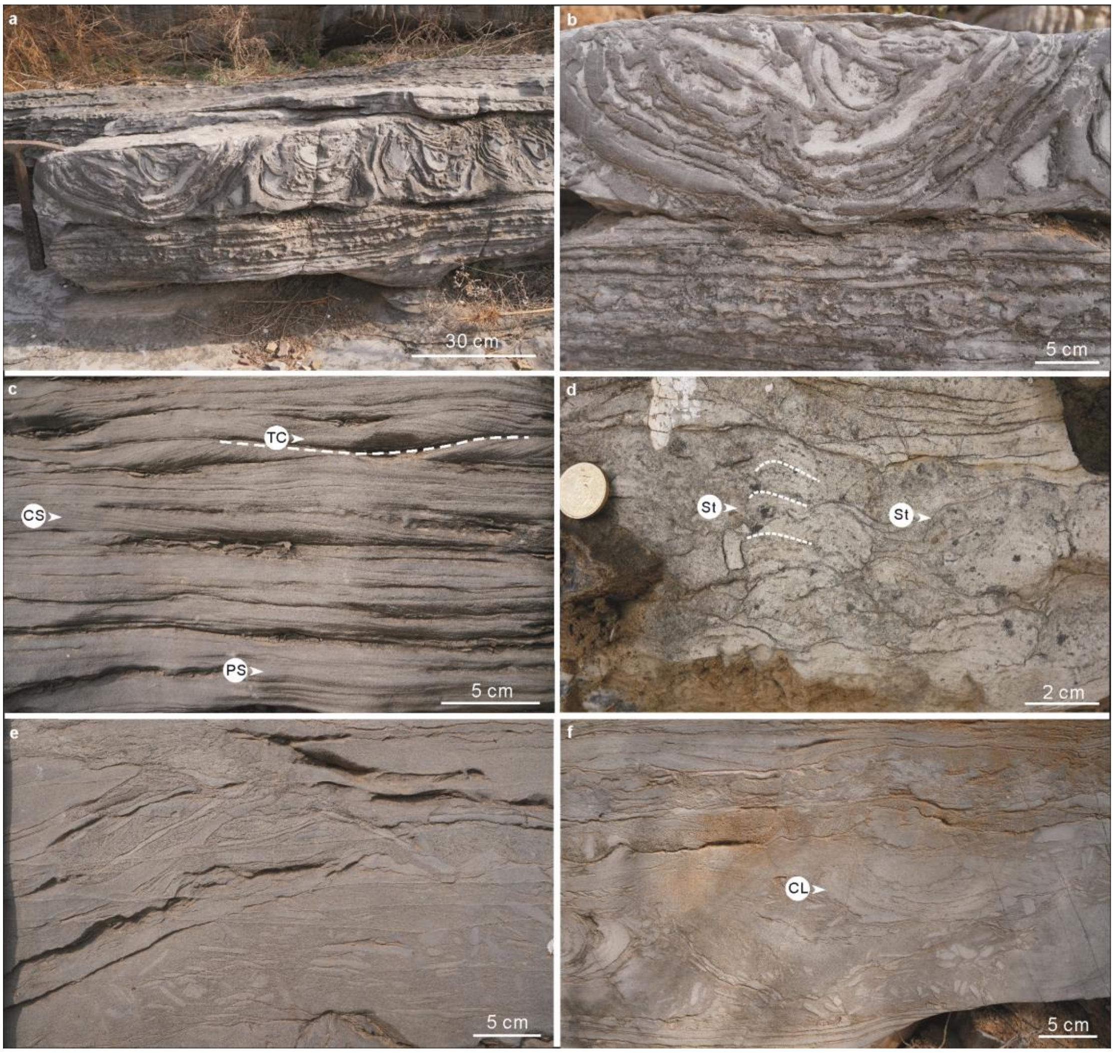

Figure S4. Illustration showing the basic morphology of *Thalassinoides* from the Cambrian of China and similar lower Paleozoic forms elsewhere. (a) *Thalassinoides horizontalis* from the late Cambrian Peerless Formation and the Lower Ordovician Manitou Formation of Colorado[4]. It was designed based on figure 6c in ref.4 by Li-Jun Zhang using CorelDRAW X4 software. Note irregular networks and presence of a thick lined wall. (b) Dominantly two-dimensional network or maze from the lower Cambrian Zhushadong Formation of North China. (c) Three-dimensional boxwork from the lower Cambrian Zhushadong Formation of North China. Note vertical shafts connecting the horizontal burrows.

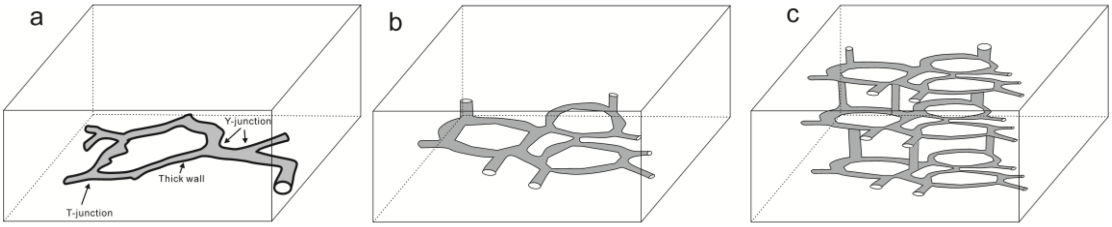

Figure S5. **Thin-section photographs of *Thalassinoides* in the Cambrian Epoch 2 Zhushadong Formation, Guankou section.** (**a–b**) Matrix consisting of micrite and *Thalassinoides* filled with dolomite. The white dashline delineates the boundary between matrix and *Thalassinoides*. Blue arrow shows small size dolomite in the matrix and yellow arrow represents large size dolomite in *Thalassinoides*. (**c–d**) Burrow and matrix stained with alizarin red. Note micro-calcite in the matrix and *Thalassinoides* filled with dolomite and some small calcite.

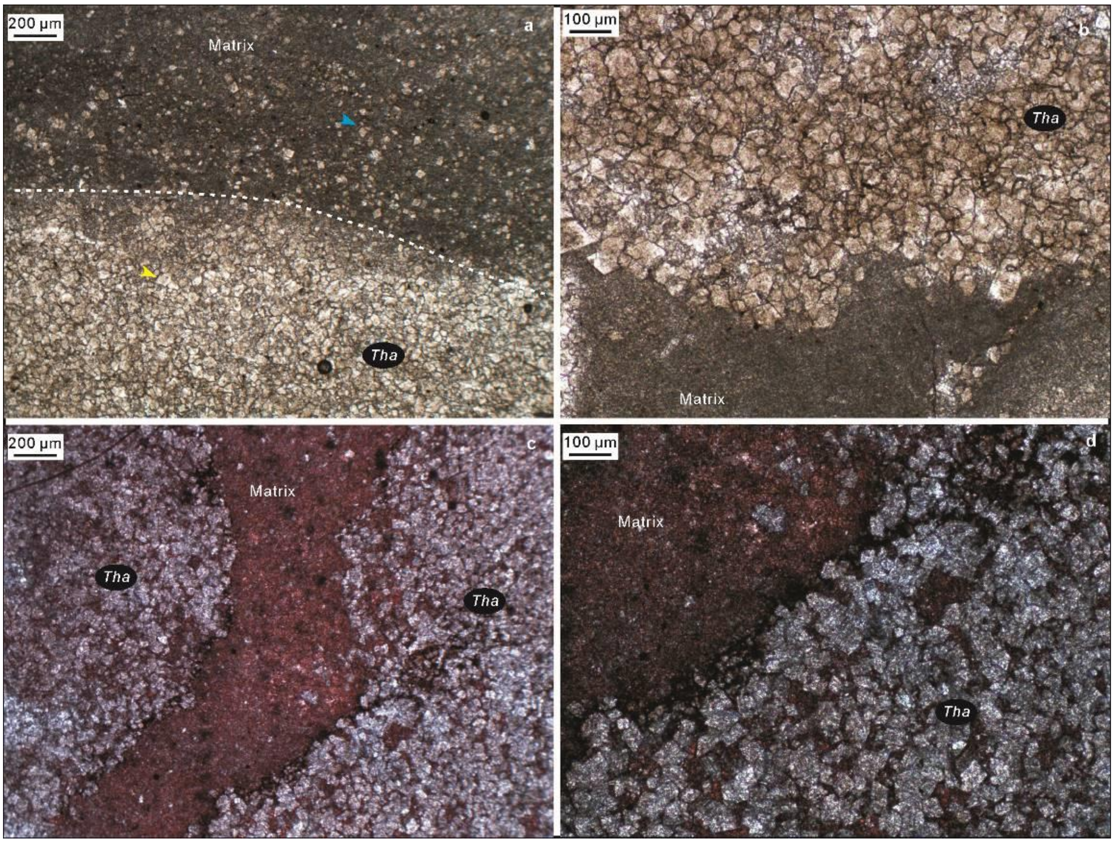

Figure S6. ***Thalassinoides* from the restricted platform deposits of the Cambrian Epoch 2 Zhushadong Formation in the Lushan section.** (**a**) General view of beds with bioturbation index 4, lower part of the Zhushadong Formation. (**b**) *Thalassinoides* networks on bedding surface, showing burrows filled with dark dolomite. (**c**) *Thalassinoides* networks on cross-section view, filling with grey marly dolomite. (**d**) T-branched *Thalassinoides* networks on bedding surface. Abbreviations: BT = Thickness of bioturbated interval, BD = Burrow depth, T = *Thalassinoides*.

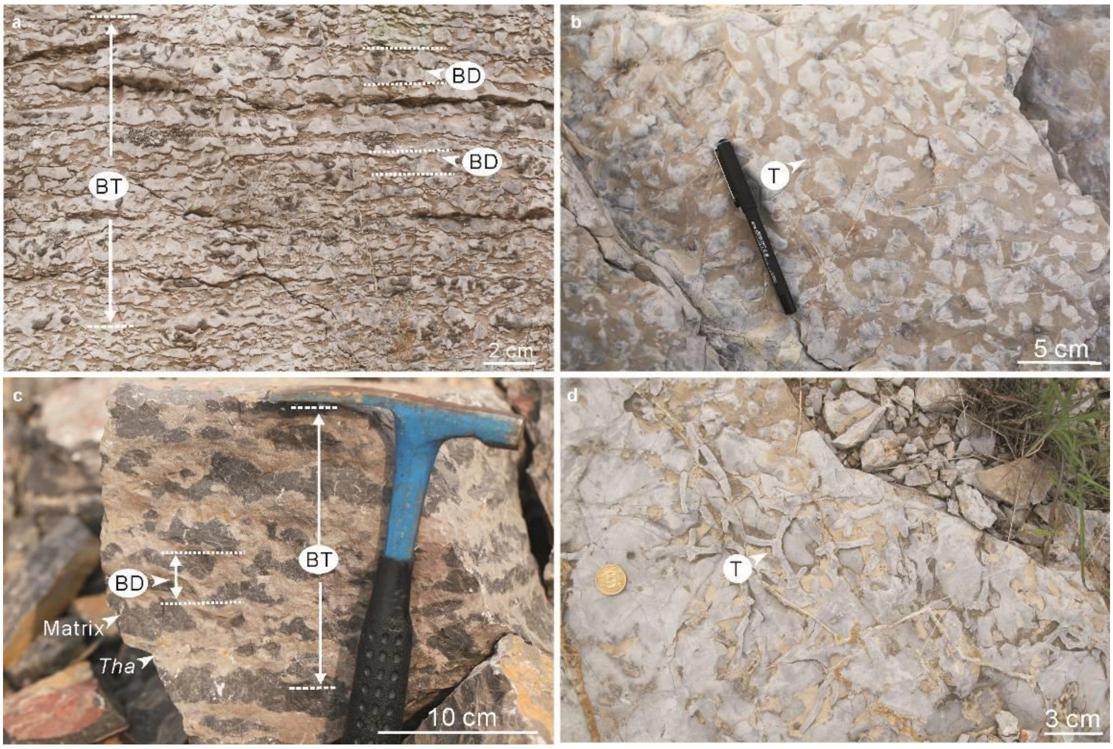

Figure S7. ***Thalassinoides* from the restricted platform deposits of the Cambrian Epoch 2 Zhushadong Formation in the Mianchi section.** (**a**) General view of beds with bioturbation index 3, lower part of the Zhushadong Formation. (**b**) *Planolites* on bedding surface co-occuring with *Thalassinoides*. (**c**) General view of beds with bioturbation index 4, middle part of the Zhushadong Formation. (**d**) Two generations of *Thalassinoides* distinguished by their cross cutting relationships, probably indicating penetration from two different colonization surfaces. Abbreviations: BT = Thickness of bioturbated interval, BD = Burrow depth, T = *Thalassinoides*, T1 = early generation of *Thalassinoides*, T2 = later generation of *Thalassinoides*.

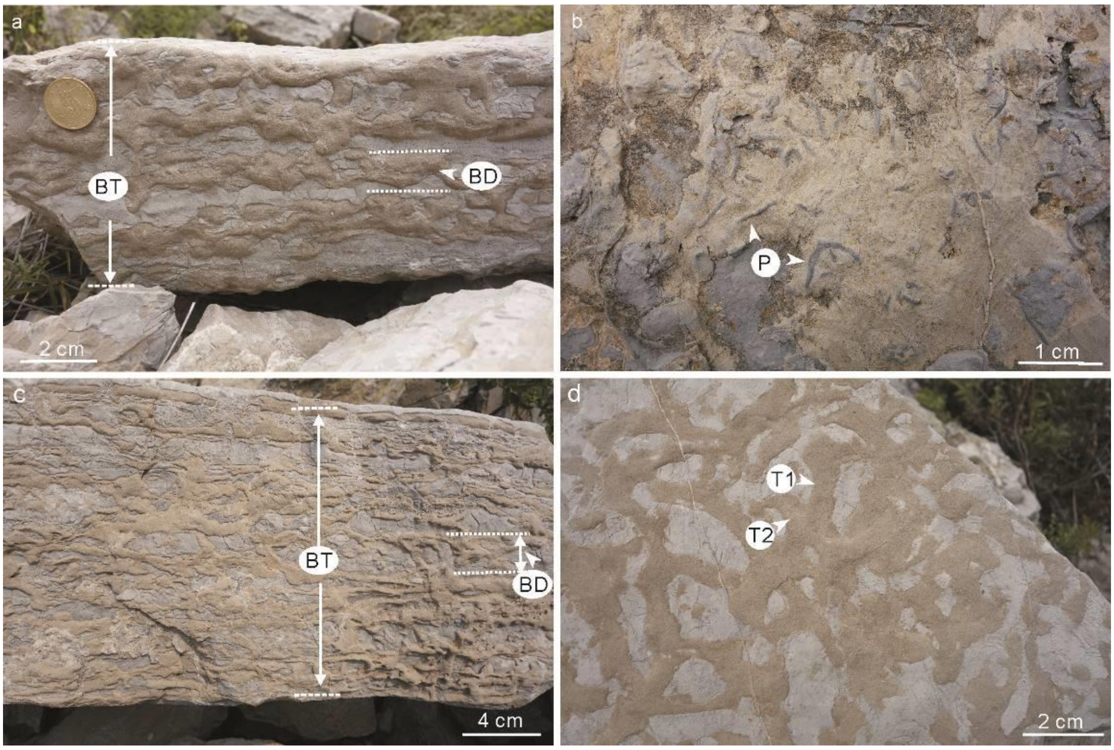

Figure S8. Generalized stratigraphic column of open platform deposits from the Cambrian Epoch 2–3 Zhangxia Formation, showing the bioturbation index and burrow depth in the Gankou section, North China.

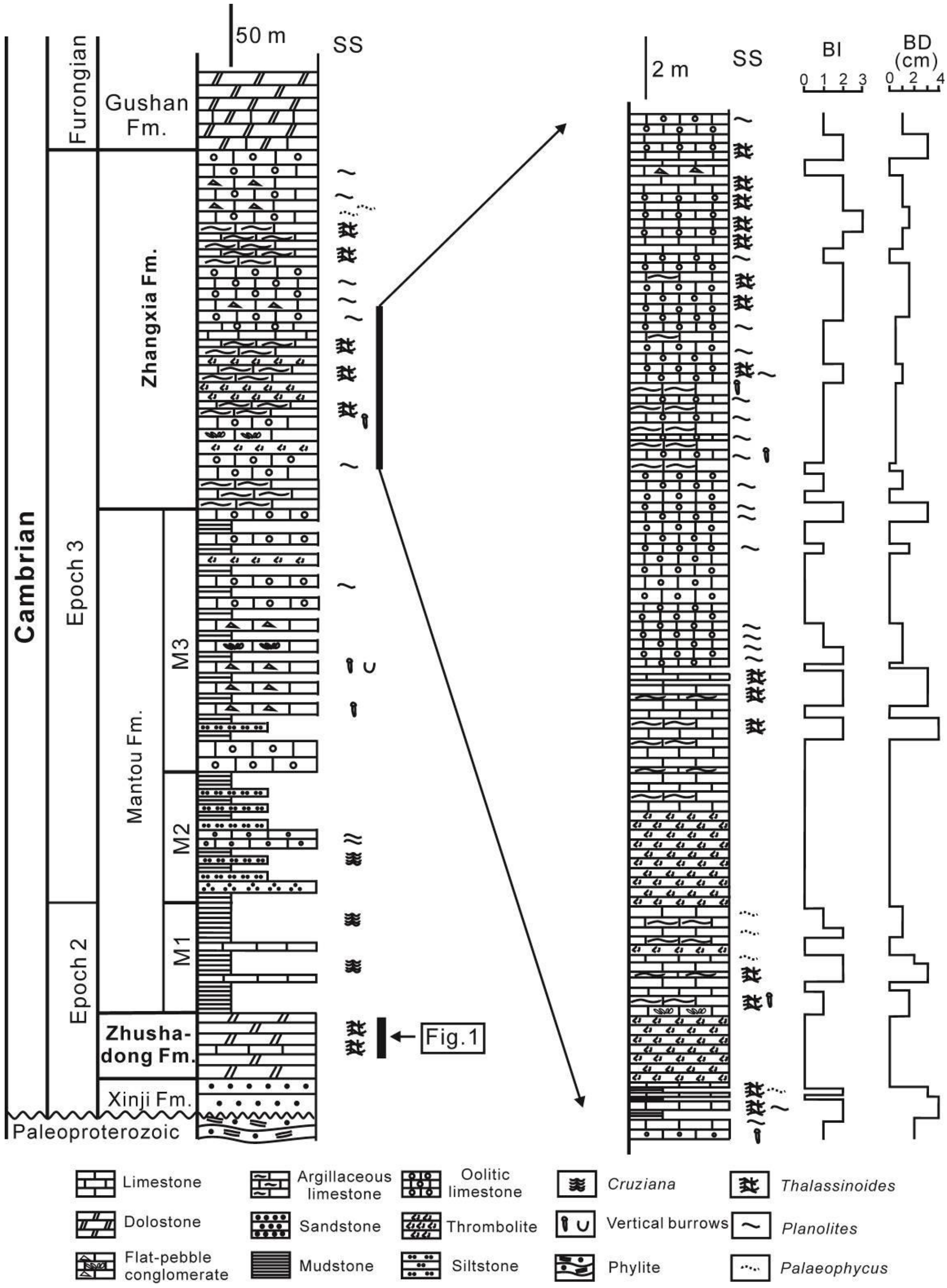

Figure S9. Trace fossils from the open platform deposits of the Cambrian Epoch 3-Furongian Zhangxia Formation in the Guankou section. (**a**) General view of beds with bioturbation index 3, lower part of the Zhangxia Formation. (**b**) *Thalassinoides* networks on bedding surface, showing burrows filled with dark dolomite. (**c**) Burrow depth in cross-section view from the middle part of the Zhangxia Formation. (**d**) *Thalassinoides* networks on bedding surface, showing burrows filled with grey marls. (**e**) Two generations of *Thalassinoides* distinguished by their cross cutting relationships, probably indicating penetration from two different colonization surfaces. (**f**) *Planolites* in oolitic limestone. Abbreviations: vs = vertical shaft, Tb = T-branched, Yb = Y-branched, BI = Bioturbation index, BT = thickness of bioturbated interval, BD = bioturbation depth, *P* = *Planolites*, T1 = early generation of *Thalassinoides*, T2 = later generation of *Thalassinoides*.

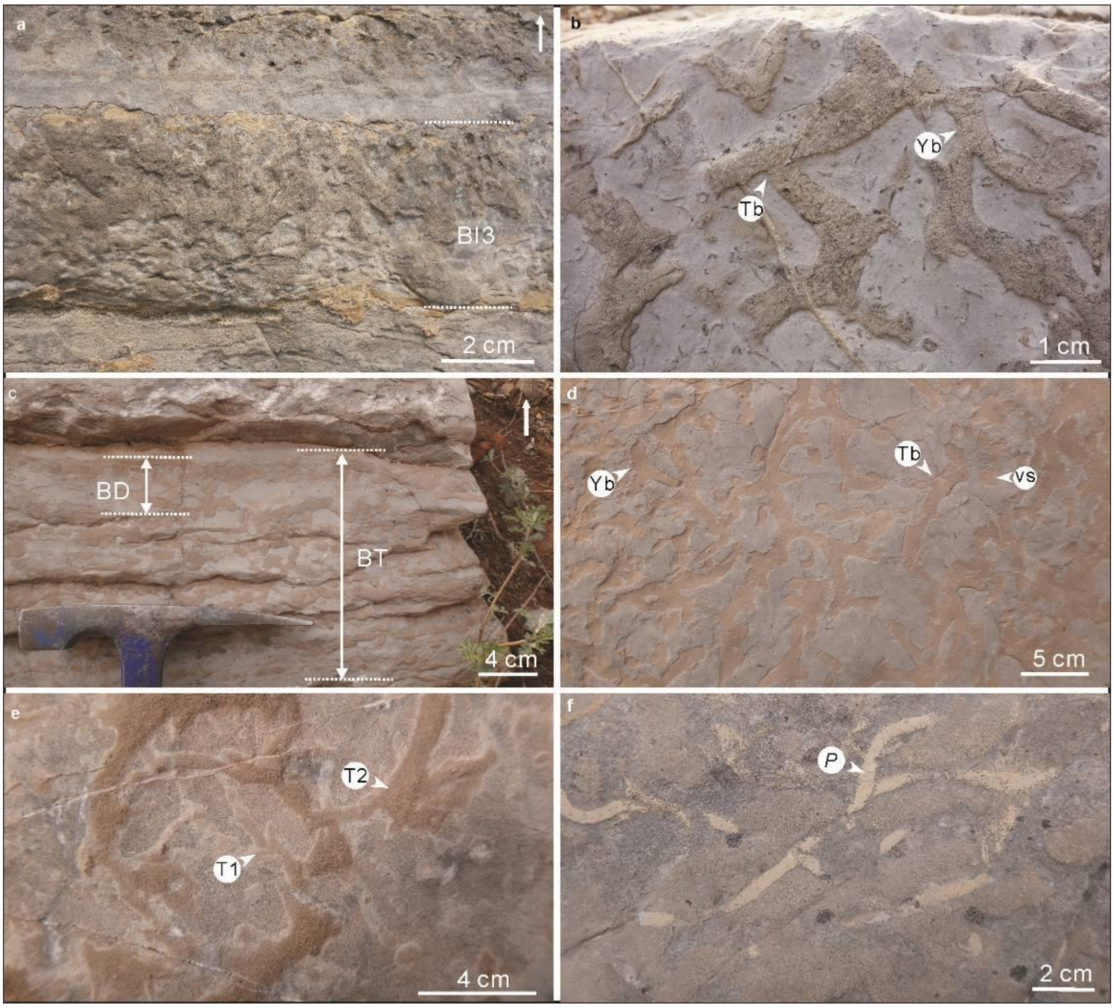

Figure S10. Sketch showing the steps for calculating the spacing between *Thalassinoides* based on the software ImageJ. (**a**) The original photo of *Thalassinoides* of the Cambrian Epoch 2 Zhushadong Formation in the Guankou section. (**b**) Draw a line (known distance) and obtain a scale (20.250 pixels/mm). (**c**) The threshold image of A using the ImageJ software. (**d–e**) Set the measurement, and then select the analyzed particles, in order to finally obtain the data of area, area fraction, and perimeter (i.e. the length of the outside boundary of the selection that have been highlighted in red using *Image>Adjust>Threshold*).

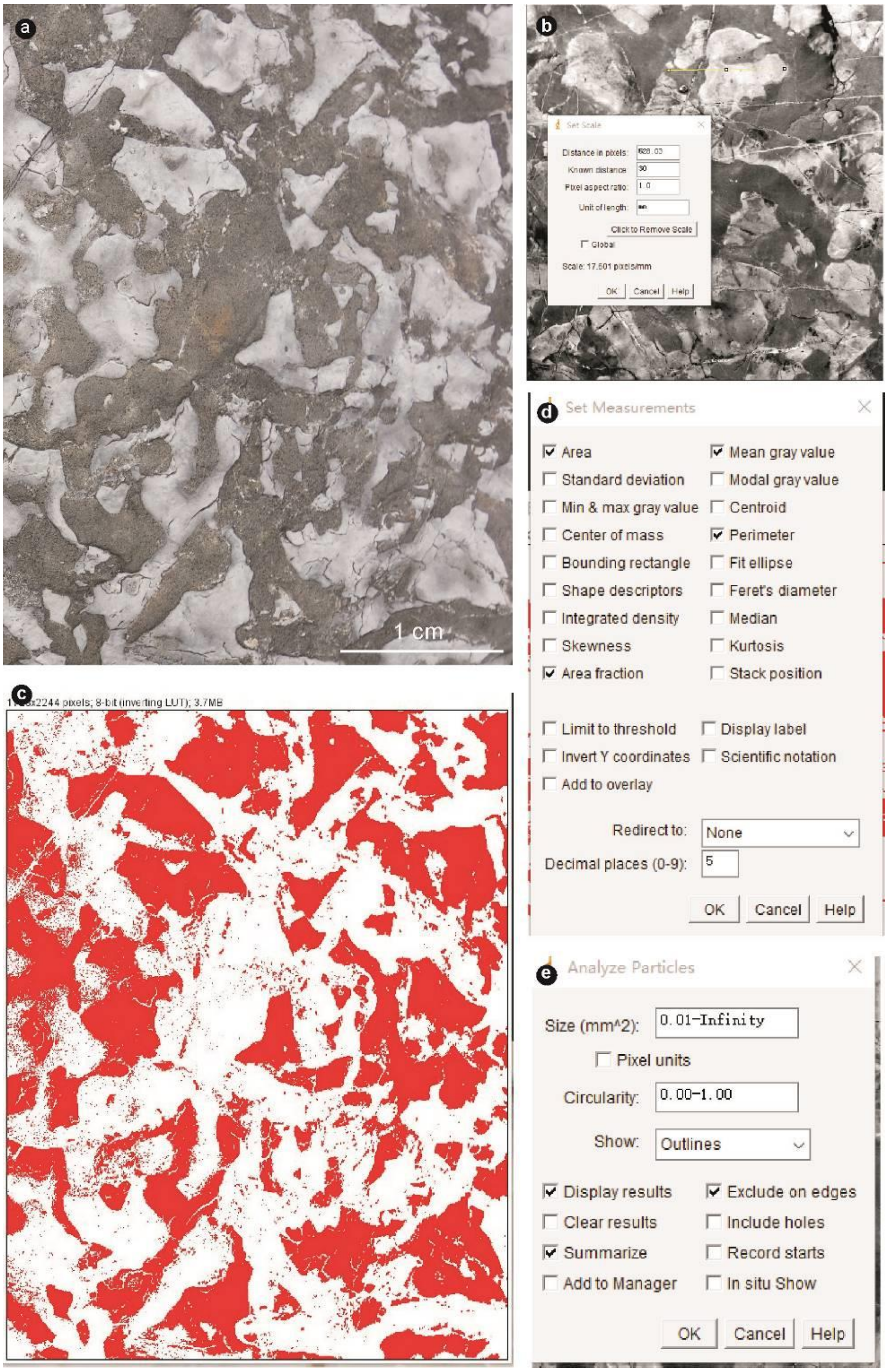

Table S1. Bioturbation index, thickness of bioturbated interval, burrow depth, and diameter of *Thalassinoides* in the Cambrian Epoch 2 Zhushadong Formation, Guankou section.

Bed Number	Bioturbation index (0–6)	Thickness of bioturbated interval (cm)	Burrow depth (cm)	Diameter of *Thalassinoides* (cm)
21	4	10	2.1	0.5–1.2
20	2	1.5	1.5	0.3–0.5
19	2	1.3	1.3	0.2–0.5
18	0	0	0	0
17	3	7	2.1	0.3–0.8
16	4	140	9.5*	0.6–1.2
15	3	14	2.2	0.5–0.9
14	4	120	24.2*	0.3–0.7
13	3	8	2.1	1–1.5
12	4	85	12.2*	0.5–0.8
11	3	8	3.1	0.3–1
10	2–4	25	9.1*	0.3–0.6
9	4	170	32.4*	0.3–0.8
8	2–5	30	5.4*	0.5
7	2–3	5	3.2	0.5–1.2
6	0	0	0	0
5	2	1.2	1.2	1.2
4	2	1.2	1.2	1.1

Table S2. Bioturbation index, thickness of bioturbated interval, burrow depth, and diameter of *Thalassinoides* in the Cambrian Epoch 2 Zhushadong Formation, Lushan section.

Bed Number	Bioturbation index (0–6)	Thickness of bioturbated interval (cm)	Burrow depth (cm)	Diameter of *Thalassinoides* (cm)
10j	0	0	0	0
10i	0–3	3.4	1.5	0.4–0.7
10h	3	8	3.1	0.3–0.7
10g	4	90	18.2*	0.6–1.2
10f	3	4	1.7	0.5–1
10e	0	0	0	0
10d	4	80	23.5*	1–1.5
10c	3	14	4.3	0.5–1.2
10b	2	1.3	1.5	0.3–0.8
10a	0	0	0	0

Table S3. Bioturbation index, thickness of bioturbated interval, burrow depth, and diameter of *Thalassinoides* in the Cambrian Epoch 2 Zhushadong Formation, Mianchi section.

Bed Number	Bioturbation index (0–6)	Thickness of bioturbated interval (cm)	Burrow depth (cm)	Diameter of *Thalassinoides* (cm)
4r	0	0	0	0
4q	2	1.5	1.5	0.3–0.5
4p	0	0	0	0
4o	0	0	0	0
4n	2	2.1	2.1	0.3–0.8
4m	0	0	0	0
4l	4	75	30.3*	0.6–1.2
4k	2	9	3.2	0.5–0.9
4j	0	0	0	0
4i	0	0	0	0
4h	4	90	22.4*	0.3–0.9
4g	3	8	2.2	0.6–1.2
4f	0	0	0	0
4e	0	0	0	0
4d	4	45	12.6*	0.3–0.8
4c	3	10	3.2	0.5–1.2
4b	2	1.1	1.1	0.9–1.1
4a	0	0	0	0

Table S4. The spacing distance between *Thalassinoides* in the Cambrian Epoch 2 Zhushadong Formation, western Henan, North China.

Section	Bed Number	Bioturbation index	Slice Count number	Spacing distance between *Thalassinoides* burrows (mm)
Guankou	10	2	1735	4.88
	17	3	1337	5.51
Mianchi	4c	3	2151	5.13
Lushan	10f	4	1895	3.03

References

1. Zhang, F. *et al*. Oxygenation of a Cryogenian ocean (Nanhua Basin, South China) revealed by pyrite Fe isotope compositions. *Earth and Planet. Sci. Lett.* **429**, 11–19 (2015).
2. Chough, S. K. *et al*. Cambrian stratigraphy of North China Platform: revisiting principal sections in Shandong Province, China. *Geosci. J.* **14**, 235–268(2010).
3. Yang, W. T., Wang, M., Qi, Y. A. Earthquake-induced Soft-sediment Deformation Structures in the Dengfeng Area, Henan Province, China: Constraints on Qinling Tectonic Evolution during the Early Cambrian. *Acta Geol. Sin-Engl*. **89**, 1835–1846 (2015).
4. Myrow, P. M. *Thalassinoides* and the enigma of early Paleozoic open-framework burrow systems. *Palaios* **10**, 58–74 (1995).
